# Hospital mortality among major trauma victims admitted on weekends and evenings: a cohort study

**DOI:** 10.1186/1752-2897-3-8

**Published:** 2009-07-27

**Authors:** Kevin B Laupland, Chad G Ball, Andrew W Kirkpatrick

**Affiliations:** 1 Department of Medicine, University of Calgary and Calgary Health Region, Calgary, Alberta, Canada; 2 Department of Critical Care Medicine, University of Calgary and Calgary Health Region, Calgary, Alberta, Canada; 3 Department of Community Health Sciences, University of Calgary, Calgary, Alberta, Canada; 4 Department of Surgery, University of Calgary and Calgary Health Region, Calgary, Alberta, Canada; 5 Regional Trauma Program, Calgary Health Region, Calgary, Alberta, Canada

## Abstract

**Background:**

Patient care may be inconsistent during off hours. We sought to determine whether victims of major trauma admitted to hospital on evenings, nights, and weekends suffer increased mortality rates. All victims of major trauma admitted to all four major acute care hospitals in the Calgary Health Region between April 1, 2002 and March 31, 2006 were included. Clinical and outcome information was obtained from regional databases. Weekends were defined as anytime Saturday or Sunday, evenings as 18:00–22:59, and nights as 23:00–07:59.

**Results:**

Four thousand patients were included; 2,901 (73%) were male, the median age was 39.5 [inter-quartile range (IQR), 22.4–58.2] years, and the median injury severity score (ISS) was 20 (IQR, 16–26). Thirty-five percent (1,405) of patients were admitted on a weekend, 30% (1,197) during evenings, and 36% (1,422) at night. Seventy-eight percent (3,106) of cases presented during the "after hours" (evenings, nights, and/or weekends). The in-hospital case-fatality rate was 447 (11%), and was not significantly different during daytime (165/1,381; 37%), evening (128/1,197; 30%), and night (154/1,422; 36%) admissions (p = 0.53), or among patients admitted on weekends as compared to weekdays (157/1,405; 11% vs. 290/2,595; 11%; p = 1.0). Admission during the after hours as compared to business hours (343/3,106; 11% vs. 104/894; 12%; p = 0.63) did not increased risk. A multivariable logistic regression model was developed to assess factors associated with in-hospital death (n = 3,891). Neither admission on weekends nor on evenings or nights increased the risk for in-hospital mortality.

**Conclusion:**

In our region, the time of admission during the day or day of the week does not influence the risk for adverse outcome and may reflect our highly developed multi-hospital acute care and trauma system.

## Background

Several studies conducted in a number of different medical, surgical, and critically ill populations have indicated that patients admitted to hospitals on weekends and evenings may suffer a higher mortality rate [1-14]. Many processes of care have been proposed to explain these observations including lower levels of staffing and restricted availability of tests and procedures during these times. In addition, physicans performance may be impaired due to shift work and fatigue during excessive work shifts or work during off-peak vigilant times [15-17]. It is also important to note that patients admitted during "after hours" may be intrinsically at higher risk for death by virtue of a different case-mix or increased severity of illness as compared to patients admitted during usual or "business" hours.

Few studies have investigated whether the presentation time for victims of trauma influences their outcome. Carmody and colleagues reviewed 8,015 major trauma admissions during 1994–1996 and found that the crude mortality rates were significantly higher among admissions that occurred at night (13.1%) as compared to during the day (10.1%), but that this was not significant after stratification by severity of illness [18]. Busse *et al *studied 1,044 trauma patients admitted to a university-affiliated level 1 trauma center and underwent surgery from 1995 until 2001 [19]. They found that the time of presentation (nighttime vs. daytime, weekend vs. weekday, month of year, and year) was not associated with in-hospital mortality after adjustment for severity of illness and other confounding variables. Arbabi *et al *analyzed 30,686 admissions to a level 1 trauma center from 1994 to 2002 [20]. After controlling for confounding variables, no difference in mortality or length of stay was observed in association with weekend or night admission.

Although after hours admissions with many medical and surgical populations are potentially associated with adverse outcomes, this may not apply to trauma patients admitted to hospitals with highly developed trauma systems. The objective of this study was to assess whether the timing of admission influences the outcome of victims of major trauma admitted to all major acute care hospitals in a large Canadian health region.

## Methods

### Patient population

This study utilized an inception cohort design. The Calgary Health Region (CHR) is a fully integrated, publicly funded health system that provides virtually all medical and surgical care to the residents of the cities of Calgary and Airdrie and a large surrounding area including smaller towns and communities (population ~1.2 million). In the CHR, adult trauma services are regionalized to the Foothills Medical Centre, and pediatric trauma services (age mandate <15 years) to the Alberta Children's Hospital. These are the only accredited tertiary care trauma centers providing trauma services for Southern Alberta, Canada (~35% of the population of the Province of Alberta). Patients may be transported for trauma care to Calgary from adjacent areas in neighbouring provinces. A small number of patients may be admitted to other general medical/surgical CHR hospitals including the Peter Lougheed Centre and The Rockyview General Hospital. All patients presenting to one of the four major CHR hospitals and registered in the Alberta Trauma Registry during April 1^st^, 2002 and March 31^st^, 2006 were included in this study. This study was approved by the Conjoint Health Research Ethics Board at the University of Calgary.

In-house, 24-hours per day coverage is provided by resident trainees or licensed physician associates. Resident coverage consists of trainees working 24 hour in-house shifts followed by mandatory time off and a maximum 1 in 4 call schedule. During the study period, trauma attending surgeons provided supervision and clinical care in one week rotations starting Friday mornings. This consisted of a 140 hour week including a continuous 82 hour call responsibility on weekends. Attending trauma surgeons are not mandated to remain in-hospital but must arrive to the hospital within 20 minutes for all serious trauma presentations and maintain a hands-on management and supervision of the trauma service. This practically translates into in-hospital call during busy trauma seasons.

### Study protocol

All study subjects were identified using the Alberta Trauma Registry that includes all patients admitted to hospital with major traumatic injury as defined by either an injury severity score (ISS) ≥ 12 or death due to trauma in the Emergency Department [21]. Detailed demographic (age, gender, location of residence, unique Alberta health number), external cause of injury (International Classification of Diseases-10^th ^Revision E-codes), ISS scores, need for intensive care unit (ICU) care, and hospital length of stay and survival status at discharge information were obtained from the Alberta Trauma Registry. Subsequent admissions to any of the CHR major acute care hospitals (Alberta Children's Hospital, Foothills Medical Centre, Peter Lougheed Centre, or Rockyview General Hospital) were obtained on all patients by a linkage with the CHR Data Warehouse using patient's unique Alberta personal health number. The institution, dates of admission and discharge, and survival status at discharge was recorded. A weekend was *a priori *defined by admissions during the period from 00:00 Saturday to 23:59 Sunday, days as 08:00 to 17:59, evening as 18:00 to 22:59, and night as 23:00 to 07:59. Where patients died prior to admission, the time of death was used as the presentation time. Business hours were defined by days (08:00 to 17:59) from Monday to Friday and the after hours was all other times (anytime Saturday or Sunday and daily 18:00–07:59).

### Data management and statistical analysis

Analysis was performed using Stata version 9.0 (Stata Corp, College Station, TX). To avoid the assessment of multiple outcomes for a single patient, only first trauma presentations were included in this study. Non-normally distributed variables were reported as medians with inter-quartile ranges (IQR) and compared using the Mann-Whitney U test. Differences in proportions among categorical data were assessed using Fisher's exact test for pair-wise comparisons and the chi^2 ^test for multiple group trend analysis. In all mortality analyses, only in-hospital deaths were assessed. A multivariable logistic regression model was developed to assess factors associated with in-hospital mortality. Variables included in the initial model were age, gender, whether the patients was transferred from another institution, admission to trauma centre vs. non-trauma hospital, ICU admission, mechanism of injury, and the following variables as determined at the admitting hospital: ISS, systolic blood pressure<90 mm Hg, endotracheal intubation, and Glasgow Coma Score <8. Backward step-wise variable elimination was then performed to develop the final model. Discrimination was assessed using the area under the receiver operator characteristic curve and calibration using the Hosmer-Lemeshow goodness of fit test.

## Results

During the four-year study period, 4,000 patients were identified with major trauma; 3,472 (87%) at the Foothills Medical Centre, 361 (9%) at the Alberta Children's Hospital, 107 (3%) at the Rockyview General Hospital, and 60 (2%) at the Peter Lougheed Centre. Seventy-three percent (2,901) of patients were male, the median age was 39.5 (IQR, 22.4–58.2) years, and the median ISS was 20 (IQR, 16–26). Thirty-five percent (1,405) of patients were admitted on a weekend, and 1,381 (35%) admissions were during 08:00–17:59, 1,197 (30%) were 18:00–22:59, and 1,422 (36%) were 23:00–07:59. More than three-quarters (3,106; 78%) of cases presented during the after hours (evenings, nights, and/or weekends).

**Figure 1:**
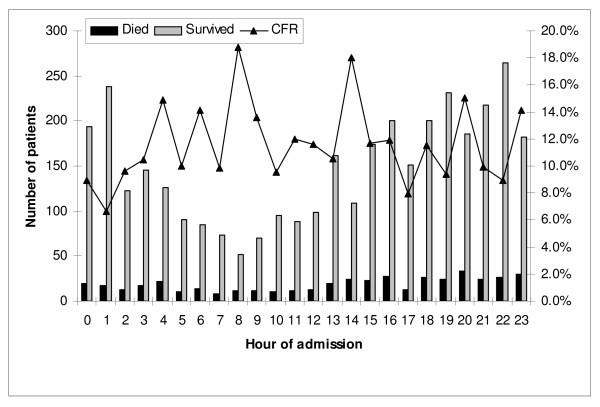
**Outcome associated with timing of admission to Calgary Health Region hospitals following major trauma (CFR; case-fatality rate)**.

**Figure 2:**
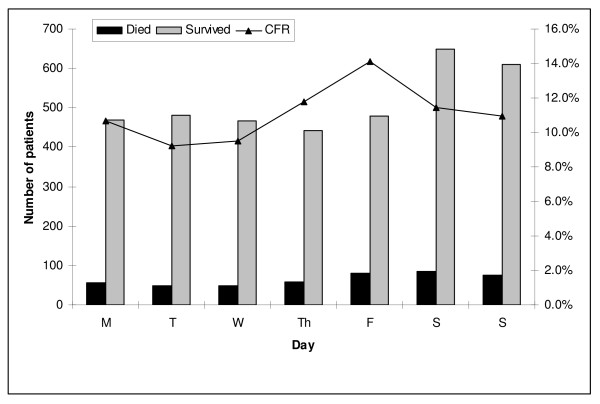
**Outcome associated with admission during the days of the week to Calgary Health Region hospitals following major trauma (CFR; case-fatality rate)**.

The in-hospital case-fatality rate was 447 (11%); 117 (3%) patients died in the emergency room and 330 (8%) died during admission to one of the four major regional acute care centres. Admissions to hospital reached a nadir at 08:00–08:59 and then the number gradually increased until a peak in the late evening/night as shown in Figure 1. The crude in-hospital case-fatality rate varied from hour to hour but no significant (p = 0.13) trend was evident (Figure 1). The case-fatality rates for daytime (165/1,381; 37%), evening (128/1,197; 30%), and night (154/1,422; 36%) admissions were not significantly different (p = 0.53). Although the case-fatality rate appeared to increase during the course of the week as shown in Figure 2, patients admitted on weekends as compared to weekdays (157/1,405; 11% vs. 290/2,595; 11%; relative risk (RR) 1.0; 95% confidence interval (CI) 0.83–1.20; p = 1.0) were not at increased risk for death. Admission during the after hours as compared to business hours (343/3,106; 11% vs. 104/894; 12%; RR 0.95; 95% CI 0.77–1.17; p = 0.63) did not increased risk.

Although no differences were observed between patients admitted during the business hours as compared to the after hours with respect to median age, gender, median length of stay, regional residency, or median ISS scores, a number of other characteristics were different as shown in Table 1. A multivariable logistic regression model was developed to assess factors associated with in-hospital death (n = 3,891, area under receiver operator characteristic curve 0.888, goodness of fit p = 1.0). As shown in Table 2, neither admission on weekends nor on evenings and nights increased the risk for in-hospital mortality.

**Table 1:** Significantly different characteristics of patients admitted with trauma during business as compared to after hours.

**Factor**	Business hours* (n = 894)	After hours* (n = 3,106)	**P-value**
Abbreviated Injury Severity Score – head	4 (2–4)	3 (1–4)	0.0028
Intensive care unit admission	244 (27%)	3106 (32%)	0.01
Admit type			0.044
Blunt	816 (91%)	2,812 (91%)	
Penetrating	23 (3%)	137 (4%)	
Burns	16 (2%)	42 (1%)	
Other/unclassified	39 (4%)	115 (4%)	
Transfer from other hospital			0.029
No	568 (64%)	1,826 (59%)	
Calgary Health Region institution	101 (11%)	425 (14%)	
Non-Calgary Health Region institution	225 (25%)	855 (28%)	
Work related	120 (13%)	213 (7%)	<0.0001
Sports injury	91 (10%)	492 (16%)	<0.0001
E-code classification			0.001
Motor vehicle traffic incidents	339 (38%)	1,216 (39%)	
Unintentional falls	287 (32%)	940 (30%)	
Assault-related	80 (9%)	261 (9%)	
Other incidents	60 (7%)	130 (4%)	
Pedal cycles	26 (3%)	94 (3%)	
Motor vehicle non-traffic incidents	18 (2%)	140 (5%)	
Suicide and attempted suicide	21 (2%)	55 (2%)	
Other road vehicle incidents	10 (1%)	72 (2%)	
Injury intention undetermined	7 (1%)	30 (1%)	
Incidents caused by fire and flame	9 (1%)	29 (1%)	
Incidents caused by drowning and suffocation	3 (<1%)	16 (<1%)	
Railway incidents	2 (<1%)	13 (<1%)	
Incidents due to natural and environmental factors	2 (<1%)	15 (<1%)	
Air and space transport incidents	0	14 (<1%)	
Legal intervention	2 (<1%)	1 (<1%)	
Water transport incidents	1 (<1%)	3 (<1%)	
Not classified	29 (3%)	78 (3%)	

* Business hours 08:00–17:59 Monday to Friday and after hours 18:00–07:59 daily and anytime Saturday or Sunday

**Table 2:** Logistic modeling of factors associated with in-hospital death among trauma victims admitted to major Calgary Health Region acute care hospitals.

Factor	Odds ratio	95% confidence interval	P-value
Endotracheal intubation	16.80	12.34–22.78	<0.001
Glasgow Coma Score <8	10.11	6.32–16.18	<0.001
Systolic BP <90 mm Hg	6.89	4.86–9.79	<0.001
Penetrating trauma	2.16	1.26–3.70	0.005
Age (per year)	1.04	1.03–1.04	<0.001
Injury Severity Score (per point)	1.03	1.01–1.04	<0.001
Transfer from other hospital			
No	1.0*	-	-
Calgary Health Region institution	0.55	0.36–0.85	0.006
Non-Calgary Health Region institution	0.41	0.30–0.56	<0.001
Weekend admission	1.02	0.79–1.32	0.862
Time of day			
Daytime	1.0*	-	-
Evening	0.99	0.73–1.33	0.924
Night	0.82	0.61–1.10	0.178

* Reference category

## Discussion

Our study conducted in four hospitals in a large health region confirms the previous work of single hospital-based studies that found no significant adjusted risk associated with time of admission and mortality [18-20]. Unlike with previous reports, we did not find any increased risk associated with admission in the evening/night in either crude or covariate adjusted analyses. In addition, we included a total of four hospitals in our study, of which two were general medical-surgical major acute care institutions. These data indicate that provision of trauma care is consistent during different times of day and days of the week in our major acute care hospitals.

The "case mix" of patients is an important determinant of outcome and variation during times of the day and days of the week and likely explains much of the after hours increased mortality effect seen in other studies to date. Similar to our observations (Table 1), several prior studies in trauma patients have documented significant differences in case-mix during different periods of the day or days of the week [18,22-26]. Controlling for diagnosis and severity of disease and case mix has reduced or eliminated the weekend effect in both prior trauma studies [18-20] and others in acutely ill non-trauma specific populations [8,10,12,13]. Adjustment for case-mix is clearly an important consideration in attempts to define consistencies in processes of care after hours.

Prior studies conducted in hospitalized populations have identified reduced staffing or decreased access to tests and procedures as potential means to explain excess mortality risk associated with off hours admissions [3,11,27]. It has been shown that adverse anaesthetics events are more common after 16:00 with inciting causes felt to be fatigue and disrupted circadian rhythms [28]. Although our trauma surgeons universally reported concerns with fatigue with the call schedule used during this study period (it has been reduced since June 2008), no association with after-hours or weekend admission and adverse outcome was shown. It is possible that the presence of trauma surgery fellows influenced after hours outcome, but given that they cover less than one-half of the call schedule and that the attending physicians typically are present for all traumas even in their presence this was likely only a minor aspect. It should be recognized that resident trainees in our centre are limited to 24 hours of consecutive clinical service. Of interest, one study has indicated that attending physicians have less cognitive skill deterioration than trainees during visiohaptic simulations to test realistic trauma interactions during fatigued conditions [15]. A final consideration from a staffing perspective is that 1–2 experienced emergency department physicians attend all initial trauma resuscitations 24 hours per day.

Although it is not uncommon to defer testing to weekdays or daytime hours in elective cases or in those where they are felt not to likely influence a patient's course, all radiological investigations, specialist consultations, and surgical procedures are available upon request 24/7 at all four of our major acute care centres. Our CT scanners are staffed 24/7 and trauma operating rooms are readily available after hours. Our intensive care units do have lower levels of staffing on weekends and evenings, and we previously found that while admission on weekends was not associated with increased risk, admission at night was after controlling for confounding variables [29]. Trauma staffing is lower in the after hours as compared to weekday daytime hours with fewer resident physicians, nursing staff, and allied health professionals such as pharmacists, physiotherapists, and respiratory therapists. Our trauma surgeons are not mandated to remain in-house but frequently do during busy trauma seasons. Studies evaluating whether having in-house trauma surgeons improves outcome have demonstrated variable results [18,30].

There are a number of important strengths and limitations of our study that merit discussion. By inclusion of all patients in a large, well-defined region, we minimized the risk of selection bias associated with conduct in a single or non-randomly selected group of patients [31]. Furthermore, external generalization of our results should be facilitated by inclusion of all ages, and admissions to both trauma-specialized and general hospitals. It is a limitation of this study that we did not have information on pre-hospital deaths as after hours pre-hospital care may have been an important determinant of outcome. Although we systematically collected detailed demographic, severity of illness, and intensity of care data and had few missing data, it is a limitation that we did not evaluate further specific underlying co-morbid illness information to allow adjustment for a range of underlying chronic illnesses. Finally, it would have been valuable to have specific staffing measures such as daily nurse to patient ratios as well as exact times to attendance by the staff trauma surgeon and the actual times of completion of investigations and consults in order to better explain our observations.

## Conclusion

We demonstrate that although the case-mix of patients varies significantly during times of day and week, this does not influence the outcome of major trauma patients admitted to our large regional acute care hospitals. While inconsistencies in care in the after hours may affect outcome of other patient populations, this does not appear to be the case for victims of major trauma, and may reflect the advanced organisation of trauma care.

## Abbreviations

CHR: Calgary Health Region; CFR: Case-fatality rate; ISS: Injury severity score; ICU: Intensive Care Unit; IQR: Inter-quartile range.

## Competing interests

The authors declare that they have no competing interests.

## Authors' contributions

KBL conceived the study, conducted the primary analysis, and drafted the article. CGB and AWK contributed to study design and interpretation of data. All authors contributed to critical revision and approval of the final manuscript.

## References

[B1] Bell CM, Redelmeier DA (2001). Mortality among patients admitted to hospitals on weekends as compared with weekdays. N Engl J Med.

[B2] Saposnik G, Baibergenova A, Bayer N, Hachinski V (2007). Weekends: a dangerous time for having a stroke?. Stroke.

[B3] Kostis WJ, Demissie K, Marcella SW, Shao YH, Wilson AC, Moreyra AE (2007). Weekend versus weekday admission and mortality from myocardial infarction. N Engl J Med.

[B4] Clark K, Normile LB (2007). Influence of time-to-interventions for emergency department critical care patients on hospital mortality. J Emerg Nurs.

[B5] Barba R, Losa JE, Velasco M, Guijarro C, Garcia de Casasola G, Zapatero A (2006). Mortality among adult patients admitted to the hospital on weekends. Eur J Intern Med.

[B6] Cram P, Hillis SL, Barnett M, Rosenthal GE (2004). Effects of weekend admission and hospital teaching status on in-hospital mortality. Am J Med.

[B7] Gould JB, Qin C, Marks AR, Chavez G (2003). Neonatal mortality in weekend vs weekday births. Jama.

[B8] Barnett MJ, Kaboli PJ, Sirio CA, Rosenthal GE (2002). Day of the week of intensive care admission and patient outcomes: a multisite regional evaluation. Med Care.

[B9] Uusaro A, Kari A, Ruokonen E (2003). The effects of ICU admission and discharge times on mortality in Finland. Intensive Care Med.

[B10] Ensminger SA, Morales IJ, Peters SG, Keegan MT, Finkielman JD, Lymp JF, Afessa B (2004). The hospital mortality of patients admitted to the ICU on weekends. Chest.

[B11] Arabi Y, Alshimemeri A, Taher S (2006). Weekend and weeknight admissions have the same outcome of weekday admissions to an intensive care unit with onsite intensivist coverage. Crit Care Med.

[B12] Weng L, Meng YL, Lu MS, Du B (2006). [Association between hospital mortality and day of week of intensive care unit admission]. Zhonghua Yi Xue Za Zhi.

[B13] Wunsch H, Mapstone J, Brady T, Hanks R, Rowan K (2004). Hospital mortality associated with day and time of admission to intensive care units. Intensive Care Med.

[B14] Luyt CE, Combes A, Aegerter P, Guidet B, Trouillet JL, Gibert C, Chastre J (2007). Mortality among patients admitted to intensive care units during weekday day shifts compared with "off" hours. Crit Care Med.

[B15] Gerdes J, Kahol K, Smith M, Leyba MJ, Ferrara JJ (2008). Jack Barney award: the effect of fatigue on cognitive and psychomotor skills of trauma residents and attending surgeons. Am J Surg.

[B16] Kahol K, Leyba MJ, Deka M, Deka V, Mayes S, Smith M, Ferrara JJ, Panchanathan S (2008). Effect of fatigue on psychomotor and cognitive skills. Am J Surg.

[B17] Pilcher JJ, Huffcutt AI (1996). Effects of sleep deprivation on performance: a meta-analysis. Sleep.

[B18] Carmody IC, Romero J, Velmahos GC (2002). Day for night: should we staff a trauma center like a nightclub?. Am Surg.

[B19] Busse JW, Bhandari M, Devereaux PJ (2004). The impact of time of admission on major complications and mortality in patients undergoing emergency trauma surgery. Acta Orthop Scand.

[B20] Arbabi S, Jurkovich GJ, Wahl WL, Kim HM, Maier RV (2005). Effect of patient load on trauma outcomes in a Level I trauma center. J Trauma.

[B21] Laupland KB, Kortbeek JB, Findlay C, Hameed SM (2005). A population-based assessment of major trauma in a large Canadian region. Am J Surg.

[B22] Zhao XG, He XD, Wu JS, Zhao GF, Ma YF, Zhang M, Gan JX, Xu SW, Jiang GY (2009). Risk factors for urban road traffic injuries in Hangzhou, China. Arch Orthop Trauma Surg.

[B23] Ricci G, Majori S, Mantovani W, Zappaterra A, Rocca G, Buonocore F (2008). Prevalence of alcohol and drugs in urine of patients involved in road accidents. J Prev Med Hyg.

[B24] Sibley AK, Tallon JM (2002). Major injury associated with all-terrain vehicle use in Nova Scotia: a 5-year review. Cjem.

[B25] Brogmus GE (2007). Day of the week lost time occupational injury trends in the US by gender and industry and their implications for work scheduling. Ergonomics.

[B26] Bugeja L, Franklin R (2005). Drowning deaths of zero- to five-year-old children in Victorian dams, 1989–2001. Aust J Rural Health.

[B27] Schmulewitz L, Proudfoot A, Bell D (2005). The impact of weekends on outcome for emergency patients. Clin Med.

[B28] Johnson J (2008). The increased incidence of anesthetic adverse events in late afternoon surgeries. Aorn J.

[B29] Laupland KB, Shahpori R, Kirkpatrick AW, Stelfox HT (2008). Hospital mortality among adults admitted to and discharged from intensive care on weekends and evenings. J Crit Care.

[B30] Luchette F, Kelly B, Davis K, Johanningman J, Heink N, James L, Ottaway M, Hurst J (1997). Impact of the in-house trauma surgeon on initial patient care, outcome, and cost. J Trauma.

[B31] Laupland KB (2004). Population-based epidemiology of intensive care: critical importance of ascertainment of residency status. Crit Care.

